# Dental Implant Rehabilitation of Posterior Maxillary Edentulism via Sinus Augmentation Using the Lateral Window Technique: A Retrospective Analysis of 289 Implants Followed Up for 15 Years

**DOI:** 10.3390/jfb16020065

**Published:** 2025-02-13

**Authors:** Alper Sağlanmak, Volkan Arısan, Cüneyt Karabuda, Hakan Özyuvacı

**Affiliations:** 1 Department of Oral Implantology, Faculty of Dentistry, Istanbul University, Fatih, Istanbul 34093, Türkiye; 2 Department of Oral and Maxillofacial Surgery, Faculty of Dentistry, Istanbul University, Fatih, Istanbul 34093, Türkiye

**Keywords:** dental implant, sinus augmentation, sinus lifting, xenograft, survival rate, complication

## Abstract

The aim of this study was to analyze the marginal bone loss and survival of implants in the augmented sinus area via the lateral window approach. The effect of sinus membrane perforation as well as splinting of the upper structure was analyzed. Two hundred and eighty-nine implants were placed in the sinus areas augmented with xenografts and collagen membranes in 101 patients. Clinical and radiographic data were obtained during recall visits. The Marginal Bone Loss (MBL) and Cumulative Survival Rate (CSR) were evaluated. The mean follow-up period was 12.4 years (range: 12 to 182 months). During the follow-up period, 19 implants were lost, yielding a 92.93% survival rate. No significant models for any of the covariates were found in terms of implant survival (*p* = 0.08). Similarly, no significant differences were observed between intact and perforated sinuses (*p* = 0.41) or between splinted or single standing implants (*p* = 0.11). The overall MBL reached 1.80 ± 0.56 mm at 15 years, and no significant differences were detected between any particular years (*p* = 0.12). Dental implant rehabilitation of the posterior maxilla via sinus augmentation using the lateral window technique is safe, effective and provides a high long-term implant survival with minimal prosthetic complications.

## 1. Introduction

Prosthetic rehabilitation of the free-ending posterior maxilla is a major challenge in dentistry. Prolonged periods of edentulism cause the enlargement of the maxillary sinus and atrophy of the crestal bone, ultimately rendering implant insertion impossible [1]. The elevation of the sinus membrane and the grafting of the created space with a suitable grafting material—known as sinus grafting or sinus lifting surgery—is utilized as the standard surgical approach, especially in cases with an inadequate residual bone height. The lateral window technique for sinus lifting was first introduced by Tatum in 1977 [2] and was initially reported to have been performed by James and Boyne in 1980 [3]. However, the complexity of surgically accessing and manipulating this area makes the clinical treatment sequence more challenging [4]. A wide range of complications have been documented in the context of the lateral window technique, including perforation of the Schneiderian membrane, sinusitis, infection of the grafted area, and mechanical and/or technical issues related to the functional loading of the implants in the grafted sinus area [5]. Less invasive techniques have been sought to mitigate the risks associated with the lateral window technique. Tatum and Summers were the first to describe the controlled fracture and elevation of the sinus floor, known as the osteotome technique, by creating microfractures in the trabecular bone [2,6]. However, this approach has resulted in challenges such as cranial bone trauma, benign paroxysmal vertigo and limited vertical bone gain [7,8]. Consequently, a variety of alternative techniques have been developed. These include minimally invasive antral membrane balloon elevation [9], piezo surgery [10], hydraulic pressure [11], and osseodensification [12]. Despite the considerable potential demonstrated by these techniques, there is a lack of long-term follow-up studies that provide robust evidence regarding their efficacy.

Several per-surgical, post-operative and post-loading complications have been reported for the rehabilitation of the posterior maxillary edentulism via sinus augmentation [13,14]. A comprehensive investigation was conducted on survival and success rates, which were investigated in relation to Schneiderian membrane perforation, the presence of preoperative sinusitis, smoking, the residual bone height [15] and the augmentation material utilized [16]. Despite the lack of consensus in the existing literature regarding the ideal grafting material, it is generally assumed that the deproteinized bovine bone mineral (xenografts) exhibits a relatively reduced resorption and infection rate [17], an increased bone density, and concomitant implant survival [18] in the augmented sinus area. However, the impact of confounding factors and potential complications on the long-term survival of dental implants and relevant prostheses remains ambiguous.

The objective of this retrospective study was to analyze the marginal bone loss and survival rate of dental implants placed in the augmented sinus via the lateral window approach, with a study period extending up to 15 years. The effect of membrane perforation and splinting of the upper structure was examined. Furthermore, complications arising in the surgical, prosthetic and follow-up phases were investigated.

## 2. Materials and Methods

### 2.1. Study Design

The study was approved by the ethical committee of Istanbul University, Faculty of Dentistry (IRB no: 2024/75), and was conducted in accordance with the Helsinki Declaration (as revised in 2013) [19]. The patient information was retrieved from the archives of the Department of Oral Implantology and the Department of Oral and Maxillofacial Surgery at Istanbul University. A detailed analysis of the registry data of patients who underwent dental implant rehabilitation via sinus grafting using the lateral window approach was conducted to identify the relevant information.

### 2.2. Patient Selection Criteria

The inclusion criterion were as follows: (1) patients who underwent panoramic imaging for implant placement with the need for maxillary sinus augmentation for the rehabilitation of the edentulous posterior maxilla, (2) patients for whom comprehensive dental implant treatment data were recorded, and (3) patients with maxillary sinus augmentation either with simultaneous or staged implant placements. The following criteria were used for exclusion: (1) patients exhibiting uncontrolled diabetes and any other systemic condition potentially interfering with bone healing, (2) patients with a history of radiation within the head and neck region during the examination and follow-up period, (3) patients with a history of intravenous bisphosphonates during the examination and follow-up period, and (4) patients with a history of heavy smoking (>10 cigarettes per day), (5) immune system disorders, (6) a chronic sinus pathology and (7) a sinus perforation ≥ 4 mm in size.

### 2.3. Premedication

The following antibiotic, analgesic, anti-inflammatory and mucosal conditioning regimen was prescribed to all patients 24 h prior to sinus grafting surgery: amoxicillin 2 g/day divided into two doses, naproxen sodium (1100 mg/day divided into four doses) and natural seawater nasal spray (one pump to each nostril 2 times a day) to prevent the risk of acute sinusitis. Patients sensitive to any of the medications were prescribed with the following alternative regimen: Clindamycin HCL (600 mg/day divided into two doses) and paracetamol (1000 mg/day divided into four doses).

### 2.4. Surgical and Restorative Steps

**Figure 1:**
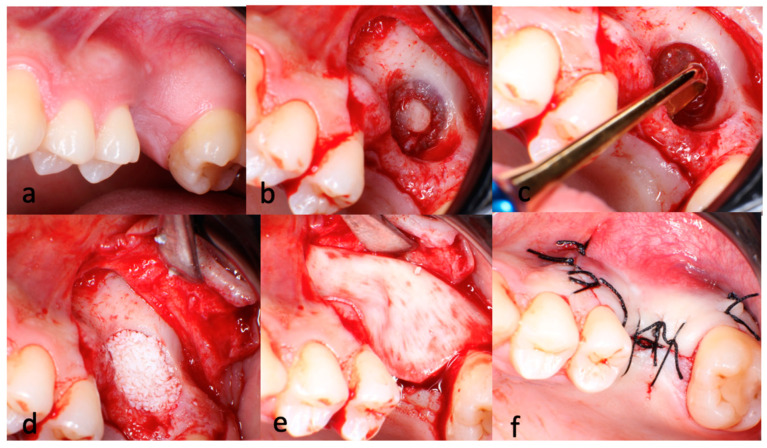
(**a**–**f**) Surgical steps of sinus augmentation via lateral window approach. (**b**) Demarcation of the lateral wall using the trap door technique. (**c**) Dissection and elevation of the Schneiderian membrane using the specially designed instruments. (**d**) Application of the graft material beneath the elevated sinus membrane (**e**) Positioning of the collagen membrane over the lateral window. (**f**) Primary flap closure with 3.0 silk sutures.

All surgical procedures were performed utilizing the lateral window approach, following the principles established by James and Boyne [3]. Briefly, oral antisepsis was established using a 0.12% chlorhexidine rinse and a full-thickness flap was raised in the edentulous area of the maxilla. The preparation of the lateral window was initiated with the utilization of a large, round steel bur and continued with a round diamond bur. Upon observing the greyish shade of the Schneiderian membrane, diamond burs were utilized for the complete demarcation of the lateral window. Care was taken to ensure that the integrity of the membrane was maintained throughout the procedure. Following the creation of an oval-shaped aperture that provided full access to the membrane, the subsequent elevation phase was commenced. Using a special set of instruments for sinus membrane elevation, the Schneiderian membrane was dissected from the sinus cavity, starting at the caudal edge and progressing mesially and distally to prevent the perforation of the sinus membrane. The membrane was then lifted vertically to allow the insertion of the dental implants to their full length (Figure 1). In cases where a sinus perforation of a size of ≤4 mm was detected [20], a resorbable collagen barrier membrane (Biogide, Geistlich Pharma AG^®^, Wolhusen, Switzerland) was sealed onto the perforation. The space created beneath the elevated sinus membrane was filled with a particulate deproteinized bovine bone mineral (Bio-oss, Geistlich Pharma AG^®^, Wolhusen, Switzerland). For perforations > 4 mm, spontaneous healing was allowed without grafting or implantation. In patients with a sufficient RBH (≥4 mm), the sinus grafting surgery and implant installation were performed simultaneously to ensure that the primary stability of the placed implants achieved a torque of >30 N/cm. Patients with a RBH of <4 mm were treated in two stages, consisting of an initial sinus grafting procedure followed by implant placement after approximately five to seven months of healing [4,21]. In each case, the lateral bone window was covered with a resorbable collagen membrane (Bio-Gide, Geistlich Pharma AG^®^, Wolhusen, Switzerland). Primary flap closure was achieved with silk sutures and a baseline panoramic radiograph was taken immediately after surgery.

After an osseointegration period of 5 to 7 months, all implants were restored with metal–ceramic prostheses following the prosthetic guidelines of C. Misch [22]. Implants were either restored as single standing units or splinted, based on individual requirements or clinical assessment.

### 2.5. Analysis of the Study Data

The patients were recalled annually, with panoramic radiographs and oral examination performed. The panoramic radiographs of the patients included in the study were digitized through scanning and subsequently transferred to a personal computer. Measurements were performed by one experienced examiner (V.A.) using a dedicated software (Dimaxis v.4.0^®^, Plandent, Helsinki, Finland). The known implant length was used as a reference and all images were calibrated prior to measurement.

The measurement of the MBL was carried out using the technique described in a previous study [23]. The distance between the implant shoulder and the most coronal point of the bone-to-implant contact was measured in the distal and mesial aspect for all implants. The average was recorded as the final value.

Per-surgical, post-op and late-term complications and adverse outcomes, including the incidence of sinus membrane perforation, infection, sinusitis, hematoma, graft and/or implant failure, porcelain chip or the fracture of the prosthesis, were evaluated.

### 2.6. Statistical Analysis

Descriptive statistics, including the mean and standard deviation (SD), were calculated. The normality of the data distribution was examined by the D’Agostino-Pearson test. A life table analysis was calculated in accordance with the methodology outlined by Prentice and Kalbfleisch [24]. The change in the peri-implant MBL between the perforated and intact sinuses over the time was analyzed using repeated measures analyses of variance and Tukey’s test for pairwise comparisons. Kaplan–Meier survival curves were generated, accompanied by a log-rank test (95% confidence interval (CI)) to evaluate and compare the survival rates of implants according to the following criteria: implants placed in the perforated and intact sinuses, and splinted and single standing implants.

To account for the effect of confounding variables (timing of implant placement (simultaneous with sinus grafting surgery or staged after 5–7 months of healing), early and late complications (implant loss, >4 mm marginal bone loss, periimplantitis), age and gender), Cox proportional hazards regression modelling was employed.

Multiple implants in one individual were addressed using variance estimators. Non-significant variables (*p* < 0.05) were excluded. Any variable identified as potentially confounding, with an associated change in the hazard ratio of 15%, was retained for further analysis.

Repeated measures ANOVA was performed using a statistical software package (Graphad Prism^®^ version 10.1.2; Graphad Software, Boston, MA, USA). Kaplan–Meier survival analysis and Cox proportional hazard regression were conducted using another statistical package (SPSS, v25, IBM Corp., Armonk, NY, USA). *p* < 0.05 was accepted as statistically significant.

Shoenfeld’s sample size formula for the Kaplan–Meier survival analysis was utilized to calculate the statistical power [25].

## 3. Results

Between the dates of May 2006 and December 2023, the data of 488 patients were accessed and a total of 387 patients were investigated on the basis of the predetermined inclusion and exclusion criteria.

Finally, the data of 101 patients who underwent sinus augmentation via the lateral window approach were included. The patient cohort consisted of 58 males and 43 females, with a mean age of 46.4 years. A total of 289 sand-blasted and acid-etched implants made by three different manufacturers (57 Camlog Implants, Camlog Biotechnologies^®^, Stuttgart, Germany, 69 Xive Implants, Dentsply Friadent^®^, Mannheim, Germany and 163 MIS Implants, Medical Implant Technologies^®^, Shlomi, Israel) were placed.

The mean follow-up time was 12.4 years (range: 12 to 182 months). The information about patients after they were last examined was used in the Kaplan–Meier estimations and labeled as “censored”.

### 3.1. Early-Term Complications and Failures

The number of reparable sinus membrane perforations (≤4 mm) detected during the surgery was 14 in 10 patients (10.29%). A total of 35 implants were placed into the perforated sinuses and the elevated space was grafted with a particulated deproteinized bovine bone mineral (Bio-oss, Geistlich Pharma AG^®^, Wolhusen, Switzerland).

A total of seven implants (2.42%) in four patients were lost during the osseointegration phase, primarily due to infection. One patient reported that the implant was found in his mouth during mastication. The remaining patients complained of pain and swelling in the implanted area, with pus being detected during the oral examination. The area was surgically exposed and the affected implants were removed following the curettage of the grafting material. At the time of suture removal, 88 (87.12%) patients showed hematoma near the area of the augmented sinus. The antibiotic prescription was extended by a period of ten days in order to prevent the onset of a subsequent infection in these patients. No acute sinusitis was observed in any of the cases. No further consequences were noted.

During the 182 months of follow-up, 12 patients (23 implants) died and a total of 20 patients (60 implants) were lost to follow-up. Twelve additional implants (4.15%) were lost in nine patients, yielding an overall survival rate of 92.93% (Table 1).

### 3.2. Marginal Bone Loss (MBL)

**Figure 2:**
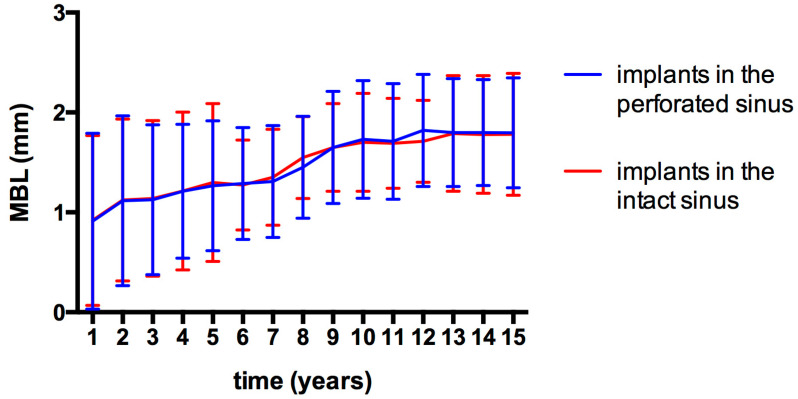
MBL around implants placed into perforated and intact sinus membrane areas. Vertical bars represent the standard deviation.

Due to drop-outs and image clarity problems, MBL measurement was only possible in 156 implants over the course of the 15-year follow-up period. Following the year of prosthetic loading, MBL values of 0.92 ± 0.88 mm and 0.92 ± 0.85 mm were recorded around the implants placed in the perforated and intact sinus areas, respectively. At the end of the 15 years, the MBL reached 1.80 ± 0.54 mm and 1.79 ± 0.58 mm. A statistically significant alteration was observed in MBL values over the follow-up period (*p* < 0.01). However, no statistically significant differences were detected between any particular years (*p* = 0.12). In addition, the variation in MBL values between the implants placed in the perforated and intact sinus areas did not reach statistical significance (*p* = 0.6); (Figure 2).

**Figure 3:**
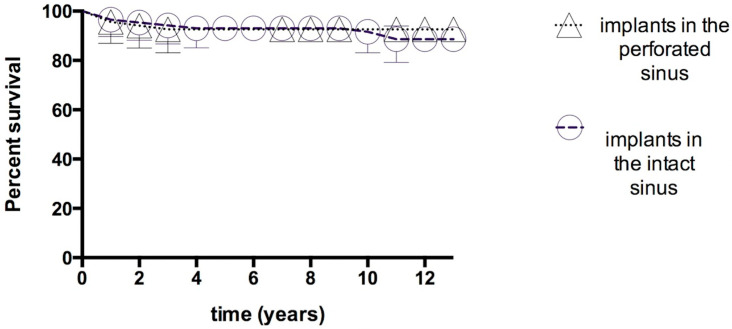
Survival curves of implant failures in the perforated and intact sinuses. Kaplan–Meier survival analysis. Log-rank test, *p* = 0.41. Symbols represent failed implants and implants lost during follow-up. Vertical bars represent the cumulative survival probability.

The investigation revealed no adverse outcomes associated with the insertion of implants into the perforated sinuses during the follow-up period. The overall survival rates of implants placed into the intact and perforated sinuses were 93.97 and 91.99%, respectively. The differences were not statistically significant (log rank test; *p* = 0.41); (Figure 3).

A total of 96 superstructures were fabricated with a splinted design to provide a higher biomechanical resistance to functional forces unless a single standing crown was insisted upon by the patient (26 single crowns in 18 patients).

**Figure 4:**
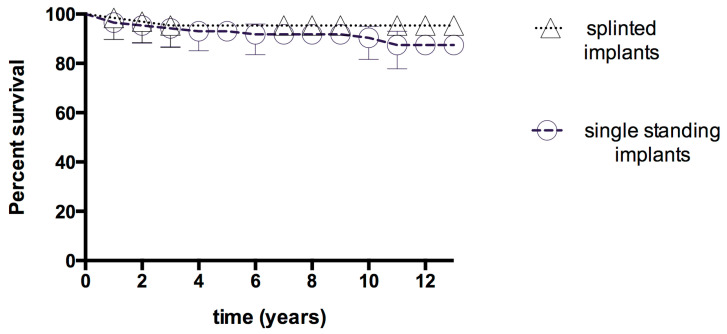
Survival curves of implant failures in the splinted and single standing implants. Kaplan–Meier survival analysis. Log-rank test *p* = 0.11. Symbols represent failed implants and implants lost during follow-up. Vertical bars represent the cumulative survival probability.

Post-implantation complications manifested predominantly as cement loosening, constituting 42.21% of the cases, and veneering porcelain chip or fracture (18.21%). At the end of the observation period, implants with splinted superstructures yielded a higher survival rate (93.11%) in comparison to implants with single standing superstructures (88.11%). However, the differences were not statistically significant (*p* = 0.11); (Figure 4).

The Cox proportional hazard regression analysis indicated no statistically significant models for any of the mentioned covariates (*z* = 11.46; *p* = 0.08). According to the resulting survival analysis data, a relative hazard ratio of 0.28 yielded a statistical power of 86% (β = 0.14).

## 4. Discussion

The long-term outcomes of sinus grafting surgery and consequent implant installation for the treatment of maxillary posterior edentulism were analyzed in this study. The exclusion criteria were strictly defined to ensure that confounding factors, such as uncontrolled diabetes and smoking, which are known to adversely affect implant survival rates, were minimized. A comprehensive analysis of a large cohort of patients and implants over a period of 15 years revealed that the lateral sinus window technique provides an adequate bone volume for the functional service of dental implants irrespective of membrane perforation or prosthetic splinting.

As demonstrated by the present findings, the survival rate of implants was found to be 92.93% over a mean follow-up period of 12.4 years. This confirms the efficacy of the surgical and prosthetic approach utilized for the rehabilitation of the atrophic posterior maxilla. The findings align with those of other studies, reporting high survival rates for implants placed in augmented sinuses [26,27]. In a systematic review, encompassing 1598 sinus lifts, Olivares et al. [28] demonstrated that the mean implant survival rate was 98%. Similarly, Flaviano et al. [29] ascertained an implant survival rate of 97.7% in 1224 maxillary sinus augmentations. The results of a comprehensive meta-analysis of long-term studies revealed that the overall patient-based implant survival rate following maxillary sinus grafting is 95% [30].

The prevalence of membrane perforation during the lateral window technique represents a notable risk, ranging from 3.6% to 58% [4,31]. A disruption of the integrity of the membrane has been proposed as a reason for the discontinuation of the procedure by some authors [32,33]. However, small perforations tend to heal spontaneously, due to the self-folding of the membrane [4,34]. Furthermore, the use of membrane repair for perforations with a diameter of ≤4 mm has been demonstrated to yield high survival rates [20]. On the other hand, large perforations carry the risk of graft migration into the sinus cavity, material loss and infection [35]. The maxillary sinus geometry and/or sinus membrane thickness may affect the integrity of the sinus membrane and introduce short and/or long-term complications like acute and chronic rhino-sinusitis [13] and oro-antral fistula [36]. The predictability of membrane repair has been reported, especially by case reports performing various modifications according to the site-specific requirements [35]. However, it is strongly advised that the perforation be treated with the application of a collagen membrane [37].

The incidence of sinus membrane perforations was low in this study (10.29%), and due to their small size (≤4 mm), they were managed effectively by sealing with a collagen membrane. Subsequent implant placements yielded a survival rate comparable to that in intact sinus membranes. This aligns with previous findings that suggest that membrane perforation does not significantly affect implant survival rates [29,34]. However, a more recent study indicated a potential increase in the incidence of implant failure, with an odds ratio of 4.125 observed in cases of perforated sinuses [27]. Similarly, Nolan et al. [38] identified a positive correlation between sinus membrane perforation and postoperative sinusitis, infection, and graft failure. Nonetheless, the dimensions of the perforations were not documented in the aforementioned studies. It was emphasized that the exact size of the membrane perforation is crucial for the success of the procedure [27]. Various clinical studies have indicated that sinus membrane perforations ranging from 2 mm to 1.5 cm can be entirely repaired without compromising bone formation or the success of the implant [39]. It has been well established that, when treated correctly, the perforated sinus membrane does not negatively impact the success and survival of the implants [5]. The success of the procedure is contingent upon a number of factors, including the lateral trapdoor technique, the placement of the window in the anterior–posterior position, the use of releasing incisions [40], and the preoperative evaluation in terms of anatomical restriction or the risk of sinonasal complications. In the present study, the trapdoor technique was employed, with the objective of preserving the cortical bone layer over the sinus. A rigorous protocol was adhered to, with premedication for sinus pathology and the continuation of antibiotics until the resolution of postoperative symptoms. Consequently, the incidence of postoperative infection was very low. The aforementioned approaches collectively reduced the perforation rate and size, allowing existing perforations to be closed with a simple collagen membrane without affecting the success of the operation.

The selection of the graft material for sinus augmentation may be important regarding the survival of the implant and longevity of the created bone volume. It has been shown that a significant reduction in graft volume can occur following maxillary sinus augmentation [16]. This phenomenon is particularly evident in instances of sinus membrane perforation, resulting in the accelerated resorption of the grafted area during the initial post-operative period [41]. It may therefore be hypothesized that selecting a graft material with a higher resorption rate could further reduce the endo-sinus bone gain. Although the resorption of the graft was not the focus of this study, there was no radiographic evidence of either the early or late loss of the vertical stability of augmented bone, even in repaired sinuses. In their systematic review, Pesce and colleagues showed that xenografts exhibited the lowest volume reduction at the early follow-up period [17]. Nevertheless, Carmagnola et al. [42] have demonstrated that sinus grafting surgery with or without biomaterial, followed by implant installation, yields comparable clinical and histological outcomes over an extended follow-up period. Certain studies have posited that the type of graft utilized is not a determining factor in the survival of the implant [43]. Instead, they emphasize the significance of the residual bone height. Furthermore, the production of deproteinized and inorganic cancellous bone components in the preparation of xenografts provides the additional benefits of preventing immunogenic graft rejection and infection [44]. The favorable outcomes observed in this study suggest that deproteinized bovine bone mineral is a viable alternative for use in maxillary sinus augmentation.

The simultaneous placement of implants in patients with extremely pneumatized maxillary sinuses is a beneficial treatment modality, as it reduces morbidity by minimizing the number of surgical interventions required [45]. In the present study, a RBH of 4 mm represents the threshold between staged and simultaneous implant installation. This stratification is crucial, as it aligns with findings from other studies indicating that adequate RBH is a significant predictor of implant success [46] and the incidence of membrane perforation [47]. In our previous investigation, a high survival rate of 95.9% was demonstrated in patients with a residual bone height of ≥5 mm who underwent augmentation and subsequent implant placement using the lateral window approach [23]. The authors emphasized the paramount importance of achieving primary stability in the residual host bone. The long-term success rate of implants was found to be elevated in cases where the residual bone height was ≥4 mm. Moreover, the perforation rate of the Schneiderian membrane was found to be relatively high in cases with a limited residual bone height [43]. Based on the present evidence, a RBH of 4 mm can be considered sufficient for achieving good primary stability and high implant survival in maxillary sinus grafting.

Recent technological developments have given rise to a range of new techniques for performing sinus augmentation less invasively through the crestal route. In a previous study [48], the osseodensification technique was employed for crestal sinus augmentation, and it was found that there was promising endo-sinus bone gain without implant failure, even in cases with a limited residual bone height of 2–4 mm. However, the study is limited by a six-month follow-up period, and further research is required to assess long-term outcomes. In a more recent multicenter study [49], 670 transcrestal sinus augmentations were performed using the osseodensification technique and simultaneous implant placement in a limited residual bone height of 2 to 7 mm. Similar perforation rates were reported (7.31%). However, it was emphasized that a perforation rate of 14.7% was observed in cases where the RBH was ≤3 mm, thus indicating that this is an important risk factor for the incidence of membrane perforation.

In the present study, the impact of particular confounding variables was examined using Cox proportional hazards regression modelling. However, no significant models were identified. Nevertheless, similar models with a variety of similar covariates were constructed. Ellegaard et al. [50] discovered that the factors affecting implant survival include the implant type, implant length, smoking, and the number of remaining teeth. In another study, the potential effects of smoking and the residual bone height on implant survival were found to be significant in a Cox proportional hazards model [51]. This discrepancy may be attributed to the limited number of complications documented during the 15-year follow-up period of this study.

MBL has been used for the objective comparison of the success of dental implants with relevance to adjunct techniques such as sinus grafting [52]. Throughout the follow-up period, MBL increased significantly over time, reaching 1.80 ± 0.56 mm at the end of the follow-up period. The absence of significant differences between the intact membrane and perforated groups in terms of MBL emphasizes that the surgical approach can be safely applied in the presence of small perforations (≤4 mm) without detrimental effects on bone stability. This finding is particularly noteworthy as it suggests that the presence of perforations may not adversely affect the long-term bone stability around implants, corroborating results from other studies [46,53]. According to the present results, the currently employed technique also provides stable MBLs in the long term for the rehabilitation of the edentulous posterior maxilla.

The complications observed during the prosthetic phase included cement loosening (42.21%), which was the most frequent issue encountered post-loading, followed by porcelain chipping or fracture (18.21%). It is evident that the aforementioned complications are contingent upon a number of factors, including the type of abutment connection, the cement used, the laboratory protocol, and the type of superstructures. Splinted and single standing implants revealed no statistically significant differences, which may be related to the relatively limited number of single standing implants in this study.

## 5. Conclusions

This long-term retrospective follow-up of 101 patients and 289 implants revealed that the utilized maxillary sinus grafting procedure is safe and effective for the rehabilitation of the posterior maxillary edentulism. A small perforation of the Schneiderian membrane (≤4 mm) has been shown to have no significant effect on the overall success of the procedure when treated with the seal of a resorbable collagen membrane. Although not statistically significant, the higher survival rate associated with splinted superstructures suggests a trend that merits further investigation from a biomechanical perspective. Further research is required to elucidate the long-term healing and durability of the xenograft volume, with a focus on MBL between instances of simultaneous and late implant placement.

## Figures and Tables

**Table 1:** Life table analysis of implants in the 15 years of follow-up.

Interval (Years)	Implants at the Start of Interval	Drop-Outs	Drop-Outs (Died)	Implants Under Risk	Failures During Interval	Survival Rate (%)	Cumulative Survival Rate (%)
0–1	289	0	0	289	7	97.58	97.58
1–2	282	1	1	280	6	97.85	95.44
2–3	274	2	0	272	2	99.26	94.71
3–4	270	1	1	268	1	99.62	94.24
4–5	267	3	3	261	0	100	94.24
5–6	261	3	9	249	0	100	94.24
6–7	249	3	3	243	0	100	94.24
7–8	243	4	6	233	0	100	94.24
8–9	233	2	0	231	0	100	94.24
9–10	231	2	0	229	1	99.56	93.81
10–11	228	3	0	225	2	99.11	92.93
11–12	223	4	0	219	0	100	92.93
12–13	219	11	0	208	0	100	92.93
13–14	208	9	0	199	0	100	92.93
14–15	199	12	0	187	0	100	92.93

## Data Availability

The original contributions presented in the study are included in the article, and further inquiries can be directed to the corresponding author.
